# Effects of hypoestrogenism and/or hyperaldosteronism on myocardial remodeling in female mice

**DOI:** 10.14814/phy2.13912

**Published:** 2018-11-14

**Authors:** Patricia Rouet‐Benzineb, Régine Merval, Evelyne Polidano

**Affiliations:** ^1^ INSERM U942 Paris Diderot University Paris France

**Keywords:** Aldosterone, calcium homeostasis, cardiac remodeling, ER stress, hypoestrogenism, redox signaling

## Abstract

We investigated the potential adverse effects of hyperaldosteronism and/or hypoestrogenism on cardiac phenotype, and examined their combined effects in female mice overexpressing cardiac aldosterone synthase (AS). We focused on some signaling cascades challenging defensive responses to adapt and/or to survive in the face of double deleterious stresses, such as Ca^2+^‐homeostasis, pro/anti‐hypertrophic, endoplasmic reticulum stress (ER stress), pro‐ or anti‐apoptotic effectors, and MAP kinase activation, and redox signaling. These protein expressions were assessed by immunoblotting at 9 weeks after surgery. Female wild type (FWT) and FAS mice were fed with phytoestrogen‐free diet; underwent ovariectomy (Ovx) or sham‐operation (Sham). Ovx increased gain weight and hypertrophy index. Transthoracic echocardiograghy was performed. Both Ovx‐induced heart rate decrease and fractional shortening increase were associated with collagen type III shift. Cardiac estrogen receptor (ER *α*, ER *β*) protein expression levels were downregulated in Ovx mice. Hypoestrogenism increased plasma aldosterone and MR protein expression in FAS mice. Both aldosterone and Ovx played as mirror effects on up and downstream signaling effectors of calcium/redox homeostasis, apoptosis, such as concomitant CaMKII activation and calcineurin down–regulation, MAP kinase inhibition (ERK1/2, p38 MAPK) and Akt activation. The ratio Bcl2/Bax is in favor to promote cell survivor. Finally, myocardium had dynamically orchestrated multiple signaling cascades to restore tolerance to hostile environment thereby contributing to a better maintenance of Ca^2+^/redox homeostasis. Ovx‐induced collagen type III isoform shift and its upregulation may be important for the biomechanical transduction of the heart and the recovery of cardiac function in FAS mice. OVX antagonized aldosterone signaling pathways.

## Introduction

Estrogens and mineralocorticoids exert their pleiotropic effects on the cardiovascular system via specific cognate receptors, the mineralocorticoid receptor (MR), and three distinct estrogen receptor (ER) subtypes, ER*α* and ER*β*, and G‐protein‐coupled estrogen receptor (GPER), which classically function as a ligand‐dependent transcription factors. Nongenomic estrogen signaling is also carried out through GPER (Barton 2012) as well as nongenomic aldosterone effects has been recently proposed to be mediated through GPER (Gros et al. 2011, 2013). Numerous reports suggest that estrogens are neuroprotective (Diotel et al. 2018) and cardioprotective (Knowlton and Lee 2012; Luo and Kim 2016) and act as anti‐oxidants that in turn sustain heart health.

Clinical and basic science studies have implicated activation of the renin‐angiotensin‐aldosterone system (RAAS), linked to the loss of ovarian estrogens, in the pathogenesis of postmenopausal diastolic dysfunction (Zhao et al. 2014). Important biological differences in heart disease determinants between sexes are now better appreciated. It is known that in hospital mortality following myocardial infarction (MI) is more than 50% higher in women than men even after adjustment for age (Milcent et al. 2007). However, the primary cause of death for women is ischemic heart disease (Roger et al. 2012). RAAS is a complex neuroendocrine system in which aldosterone is responsible for the homeostasis of water and electrolytes (sodium and potassium) and plays a pivotal regulator of cardiovascular function such as blood pressure control (Hermidorff et al. 2017). Chronic activation of RAAS leads to hypertension as well as proinflammatory, ‐thrombic and –atherogenic pathways. Experimental and clinical studies report that elevated circulating aldosterone promotes cardiac hypertrophy, fibrosis, ventricular arrhythmia and cardiac dysfunction (Duprez et al. 2000; Rocha and Funder 2002). Despite these detrimental effects of aldosterone, the Randomized Aldactone Evaluation Study (RALES), Eplerenone Postacute Myocardial Infarction Heart Failure Efficacy and Survival Study (EPHESUS) and EMPHASIS clearly demonstrated that antagonizing aldosterone receptor are effective treatment strategies although gender differences in efficacy of treatment are not routinely determined (Pitt et al. 1999, 2003; Zannad et al. 2011).Data from the Framingham Heart Study suggest that women may be more sensitive to the deleterious cardiovascular remodeling effects of aldosterone (Vasan et al. 2004). Recent studies show that excessive catecholamine levels due to emotional and physical stress triggered Tako‐Tsubo cardiomyopathy, a reversible form of acute heart failure, in elderly postmenopausal women (Ueyama 2004; Nef et al. 2009; Schneider et al. 2013). Estrogen deficiency may be involved in this myocardial disease. The mechanisms by which estrogen loss affect catecholamine‐ stressed heart are also uncertain. There is evidence that heart failure differentially impacts male and females (Blenck et al. 2016), and that estrogen can modulate the transcriptional activity of MR (Barrett Mueller et al. 2014). The MR genotype moderates the influence of estrogen and progesterone on emotional information processing (Hamstra et al. 2015, 2016, 2017). Catecholamines, glucocorticoids and, first of all aldosterone, constitute a stress‐induced hormonal “cocktail” responsible for the stress‐dependent and then for sustained blood pressure elevation (Antonov et al. 2016). Therefore, aldosterone is now considered as emotional stress hormone which plays an important contribution to the pathogenesis of stress‐dependent arterial hypertension (Antonov et al. 2011).

Although there are studies that demonstrated the deleterious effects of either estrogen deprivation or excess of aldosterone, there is still a paucity of study regarding the effect of estrogen deprivation and hyperaldosteronism on cardiac remodeling and function. Therefore, using a murine hyperaldosteronism mouse model developed by our laboratory, to test combined effects of hypoestrogenism induced by ovariectomy and excessive aldosterone level in nonstressed heart deserve to be investigated.

In our transgenic mouse hyperaldosteronism model (Garnier et al. 2004), mice of both genders are healthy with no apparent morphological abnormalities. However, male mice overexpressing cardiac aldosterone synhase (AS) exhibit a coronary endothelial dysfunction due to the altered potassium channel BK_Ca_ expression and activity, only (Ambroisine et al. 2007). The high aldosterone exerts its deleterious cardiovascular effects via MR stimulation and worsens cardiac fibrosis by repressing B‐type natriuretic peptide (BNP) and bone morphogenetic protein 4 (BMP4) in hypertensive mice (Azibani et al. 2012a) and by inhibiting the reexpression of fetal genetic program (*β*‐myosin heavy chain: *β*‐MHC, and atrial natriuretic peptide: ANP) (Azibani et al. 2012b). Besides fibrosis, aldosterone regulated 265 cardiac genes (Messaoudi et al. 2013) and induced the proliferation of cardiac endothelial cells (Gravez et al. 2015).

To our knowledge, the activation of Ca^2+^‐signaling and SR/ER stress pathways of LVH in AS overexpressing mice, and whether this activation displays hormonal differences has not been investigated. Like the endoplasmic reticulum (ER), the sarcoplasmic reticulum (SR) which is considered as a reservoir for Ca^2+^ release via the RyR2 channel during systolic contraction, then for Ca^2+^ capture via SERCA2a during relaxation, can also be stressed, either by altered SR Ca^2+^‐homeostasis and/or by altered proteins which trigger cardiac dysfunction.

Accordingly, our aim was to assess the contextual modulation of estrogen loss and aldosterone‐stimulated signaling pathways on Ca^2+^/redox‐homeostasis, especially the balance between pro‐ (CaMKII, calcineurin (Cn)/NFAT) and antihypertrophic (GSK3*β*), SR/ER stress (GRP78, PDI, and CHOP) and pro‐ (Bax, Caspase 3) and antiapoptotic (Bcl2), prooxidant (eNOS, Nox4, Gp91‐phox) and antioxidant (Nrf2, MsrA) signaling pathways, in relation to calcium‐handling proteins in AS‐overexpressing female mice.

## Materials and Methods

### Ethical approval

The transgenic mice overexpressing aldosterone synthase in heart, on a FVB background (the 8282M line), have been described in detail previously (Garnier et al. 2004) and were produced in CDTA (TAAM‐CNRS, Plateforme Animalerie, Orleans UPS 44, France). Female wild type (FWT) and mice overexpressing aldosterone synthase (FAS) were used. Mice were housed in a specific pathogen‐free facility and handled in accordance with European Union Directives (86/609/EEC) on care and use of laboratory animals. The review and approval of the study was obtained by the local Animal Ethics Committee (ref: 04346.01).

### Experimental design

#### Bilateral ovariectomy

Female wild‐type (20) and transgenic (20) mice from the same litters were housed 2 per cage, under a 12/12 h light dark cycle at 21°C and allowed free access to water and phytoestrogen‐free diet (Teklad 2019, Harlan, Belgium) from the beginning of the experiment.

Animals (40) were then subdivided randomly into two groups, either sham‐operated (ovaries were exteriorized, but not removed; Sham), or ovariectomized (bilateral ovariectomy, Ovx). Briefly, 15 min before surgical procedure, mice received buprenorphine (100 *μ*g/kg) injected subcutaneously. Four–five week‐old female mice weighing 18–22 g, were anesthetized using a mixture of ketamine/xylazine solution (160 mg/kg, 6 mg/kg, respectively) injected intraperitoneally. A 1‐cm incision was made in the skin and back muscles parallel to the midline of the animal. Next, the ovaries were located, and the oviduct, including the ovarian blood vessels, was ligatured, followed by ovary removal. The incision in the back musculature was closed with an absorbable thread (Ethicon 5.0) and the skin was sutured with the same thread. Throughout the surgical procedure, mouse body temperature was maintained at 37°C with a heating pad.

#### Experimental groups

Experimental groups consisted of four groups (with 10 mice per group): female mice of the two genotypes were submitted either to surgery (ovariectomy [Ovx] or sham‐operation (Sham**).** A total of 40 female mice were grouped into FWT‐Sham or FAS‐Sham and into FWT‐Ovx or FAS‐Ovx. Echocardiography was used to follow cardiac function and was performed in the morning. Mice were killed with lethal pentobarbital ip injection at 9 weeks after surgery. Immediately, the organs (heart, lung, liver, and uterus) were removed and weighted, tibia length was measured. Hearts were quickly excised; then chambers (atria, ventricles) were dissected, weighed, and immediately frozen in liquid nitrogen and stored at −80°C until use.

#### Echocardiography

Before transthoracic echocardiography, mice were preanesthetized with 1% isoflurane in 1 L oxygen per minute in induction chamber (isoflurane vaporizer, Havard apparatus). Mice were placed in the supine position on a heated pad and anesthesia was maintained via a face mask by continuous isoflurane ventilation (0.5–0.8%) on a mixture of room air and oxygen. Isoflurane was titrated to maintain heart rate between 450 and 600 beats per minute. Echocardiograms of the left ventricle were obtained using an ACUSSON S3000 (Siemens, Healthcare, Germany), device equipped with 14 MHz linear transducer. M‐mode recordings were obtained of the left ventricles in the parasternal short axis view at the level of the left ventricular papillary muscles. Five consecutive cardiac cycles were measured; the mean value of these cycles was calculated. Measurements were made of the left ventricular internal dimension in diastole and systole (LVEDD, LVESD), and left ventricular posterior wall thickness in diastole and systole (LVPWd, LVPWs). These measurements were then used to calculate the left ventricular fraction shortening (FS) as previously described (Vergaro et al. 2016).

#### Systolic blood pressure measurement

Using a programmable tail‐cuff sphygmomanometer (CODA, noninvasive blood pressure system; Kent Scientific Corporation, Torrington, CA), systolic, diastolic, mean blood pressure, and heart rate were measured noninvasively in vigil mice of different experimental groups. In order to minimize their stress, the animals were trained in constraint conditions for two weeks before the systolic blood pressure (SBP) measurement.

#### Histological evaluation

Masson's Trichrome staining kit (Réactifs RAL, France) was used to quantify myocardial fibrosis in mice surgery with or without surgery. Frozen sections of heart (7 *μ*m) were taken and were successively stained with Mayer Haemalun (10 min), rinsed with water, then stained with Ponceau Fuschin solution (5 min), rinsed in two bats of 1% acetic water. The staining was fixed in a bath of phosphomolybdic acid (3 min) and immediately stained in aniline blue solution (5 min) following by 2 baths of 1% acetic water. They were dehydrated by successive baths in alcohol (60–100%). The slides were dipped in a bath of xylene and mounted with synthetic resin (Eukitt). The collagen fibers were stained blue and the nuclei stained black and the cytoplasm was stained red. Ten images of 5 sections per animal of each group were taken under the light microscope at ×20 magnification. Using a semiautomatic image‐analysis system (Microvision, Lisses, France) linked to a light microscope; histomorphometry was conducted to quantify myocardial fibrosis, cardiomyocyte area. The results are expressed as the myocardial fibrosis and calculated as the ratio of the fibrotic area (blue) to the entire heart area (collagen fiber [blue] and myocyte area [red]). LV cardiomyocyte area (*μ*m^2^) was assessed. One hundred or more cardiomyocytes showing a central nucleus were randomly selected and areas were measured from each section per animal.

#### Biochemical analyses (hormonal measurements)

Blood was collected into EDTA‐treated tubes from the heart during the euthanasia then plasma was isolated by centrifugation at 2000*g* for 15 min at 4°C. The resulting supernatant is designated plasma. Following centrifugation, the plasma was immediately transferred and was aliquoted into a cold polypropylene tube, stored at −20°C or lower temperature to avoid freeze‐thaw cycles. Aldosterone plasmatic concentrations were measured by radioimmunoassay (RayBiotecth company) and plasma 17‐*β* estradiol (E2) measured by ELISA kit (Cayman company) according to the manufacturer's instructions.

#### Western blot

Frozen cardiac ventricles from mice were individually pulverized in liquid nitrogen, to yield a fine powder. The powder was homogenized in lysis buffer (20 mmol/L TRIS‐HCl, pH 7.4, 150 mmol/L NaCl, 1% TritonX‐100, 1 mmol/L EGTA, 1 mmol/L EDTA, 0.5% sodium deoxycholate) supplemented with a protease inhibitor cocktail (Sigmafast), and the phosphatase inhibitor cocktails 2 (P5726) and 3 (P0044) from Sigma‐Aldrich, then solubilized at 4°C for 30 min. The LV homogenates were centrifuged at 10,000*g* at 4° C for 30 min. Protein concentrations of the supernatants were determined by the Bradford method, and were in the range of 10–15 *μ*g/*μ*L. Twenty *μ*g proteins of each cardiac LV homogenate were separated by a 4–20% gradient, 10 or 18% SDS‐PAGE, and transferred onto nitrocellulose membranes. Transfer efficiency was checked by Ponceau S staining (P3504, Sigma Aldrich, France). Membranes were blocked then blotted with diluted primary antibodies. Source of primary antibodies : anti‐MR (ID5, r‐MR1‐18) was a generous gift from Dr Celso Gomez‐Sanchez (Endocrine Section, G. V. (Sonny) Montgomery VA Medical Center, Jackson, MS, University of Mississippi Medical Center, Jackson, MS)., from Cell Signaling technology [anti‐ Pan‐Calcineurin A, CST #2614; anti PDI (C81H6), CST#3501; anti‐p38 MAPK, CST#9212; anti‐phospho‐p38MAP kinase (Thr180/Tyr182), CST #9211; anti‐caspase 3, CST #9662; anti‐Phospho GSK3*β*(pSEr9), CST#9336; anti‐ phospho‐p44/42 MAP kinase (Thr202/Tyr204) (ERK1/2), CST #9101; anti‐p44/42 MAP kinase, CST#4695**;** anti–phospho Akt (Ser473), CST #9271; anti‐Akt, CST #9272; anti–phospho GSK3*β* (Ser9) CST#9336); anti‐GSK3*β* (27C10), CST#9315: anti‐eNOS, CST#9586;anti‐PDI (C81H6), CST#3504; anti‐CHOP (D46F1), CST#5554]; Abcam [anti‐collagen I – C‐terminal (ab 209539); anti‐collagen III (ab7778); anti‐SERCA2 ATPase, (ab3625); anti‐GRP78 BiP, (ab21685); anti‐calcipressin1 (MCIP1), (ab140131)]; Santa cruz biotechnology **[**anti‐ gp91‐phox (C15), sc‐5827; anti‐ gp91‐phox (54.1), sc‐130543; anti‐NF*κ*Bp65 (C‐20), sc‐372; anti‐MsrA(5B5), sc‐59620**;** anti‐pBcl2 (p‐Ser 87)(C‐2), sc‐377576; anti‐Bax (6A7), sc‐23959; anti‐NFATc4 (C20), sc‐1153; anti‐phospho–NFATc4 (p‐Ser168/170), sc‐32630; anti‐ER*α* (C‐311), sc‐787; anti‐ER*β*, sc‐390243**;** anti‐ NAPDH oxidase 4 [Nox4 (L‐20)], sc‐55142; anti‐Nrf2 (C20) sc‐722; anti phospho‐c‐Jun p39 (p‐Ser63) sc‐822; anti‐c‐Jun (H79) sc‐1694]; Swant [anti‐NCX, R3F1]; Badrilla **[**anti–phospho PLN (Thr17), AO10‐13; anti–phospho PLN (Ser16), AO10‐12; anti‐PLN, AO10‐14]; Southern Biotech [anti‐Bcl2, #1006501]; Millipore [anti –GAPDH, clone 6C5, MAB 374; anti‐oxidized –CaM Kinase II (Met281/282), #07‐1387]; ThermoFisher Scientific [anti‐RyR1 (34C), MA3‐925; anti‐PLN (2D12), MA3‐922; anti‐MsrA PAS‐14206; anti‐phospho‐CaMKII(Thr284), MA1‐047; anti‐CaMKII*δ*, Pa(622168]. After three washes, they were finally incubated with horseradish peroxidase (HRP)‐conjugated anti‐mouse, anti‐goat or anti‐rabbit secondary antibodies, as appropriate. Visualization by chemiluminescence was carried out according to the manufacturer's instructions (ECL kit, GE Healthcare) and recorded using AZURE C500 digital imager (Sciencetech, France), time exposure was 45 seconds. Equal protein loading for LV lysates was assessed by stripping and reprobing blots with an anti‐GAPDH antibody). Quantification of digitized images of immunoblots was done using ImageJ software [http:/rsb.info.nih.gov/nih‐image/about.html.]. The intensity of immunoreactive bands was normalized to that of GAPDH. Data are expressed as means ± SEM.

#### Statistical analysis

Data are expressed as means ± SEM. The measured LV parameter analyses were evaluated, using one‐way analysis of variance (ANOVA) for multiple comparisons and followed by group‐to‐group comparison with the Bonferroni correction as post hoc test using GraphPad Prism 5 statistical software. A *P* ≤ 0.05 was considered statistically significant. The *n* values in the legends indicate numbers of independent biological samples used for the analyses.

## Results

### Subtle changes in macroscopic myocardial structure and function

**Figure 1:**
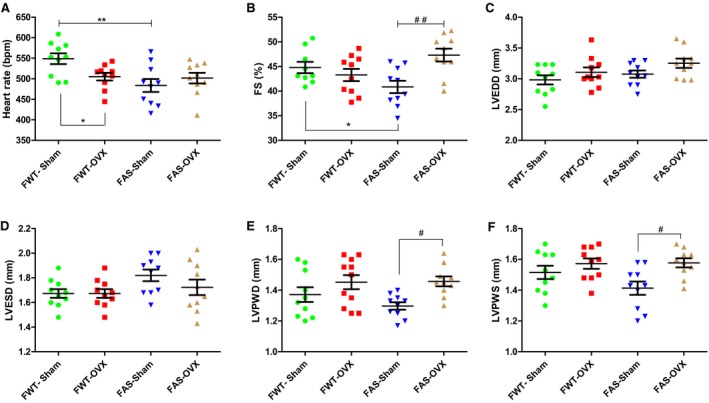
Echocardiographic parameters of functional and structural characteristics of the sham‐operated (Sham) or ovariectomized (Ovx) female wild type (FWT) or overexpressing cardiac aldosterone synthase (FAS) mouse hearts. (A) Heart rate (beat per minute: bpm); (B) Fractional shortening (FS) was calculated from 10 animals of each experimental group and expressed as percentage; (C,D) left ventricle (LV) dimensions at diastole (LVEDD) and at systole (LVESDs), (E,F), left ventricular posterior wall thickness at end diastole (LVPWd) and at en systole (LVWPs) from the same animals are expressed in millimeters. All data are the mean ± SEM. *P < 0.05 versus FWT‐Sham, and **P < 0.01 FAS‐Sham versus FWT *‐*Sham, ^#^ *P* < 0.05 FWT‐Ovx versus FAS‐Sham, ^##^ *P* < 0.01 FAS‐Ovx versus FAS‐Sham.

Echocardiographic parameters for left ventricle structure and systolic function are reported in Table 1 and shown in Figure 1. Heart rate was lower in FAS‐Sham than in FWT‐Sham mice whereas they were comparable between ovariectomized FWT and FAS mice. However, FWT‐Ovx mice exhibited a significant slight decrease in heart rate (Fig. 1A). Fractional ventricular shortening (FS) was unchanged between FWT mice (Sham or Ovx) (Fig. 1B). No significant differences in LVEDD or LVESD were observed comparing the groups (Fig. 1C and D). Ovx significantly induced a slight increase in FS (+10.7%, *P* = 0.006) (Fig. 1B) and in left ventricular posterior walls (Fig. 1E and F) in FAS mice, only.

**Table 1:** Echocardiographic parameters of cardiac structure and function

Genotype	FWT		FAS	
Surgery	Sham	Ovx	Sham	Ovx
Number of mice	10	10	10	10
HR (bpm)	549 ± 13	505 ± 9	483 ± 16^a^	501 ± 12
LVEDD (mm)	2.9 ± 0.1	3.1 ± 0.04	3.1 ± 0.1	3.3 ± 0.1
LVESD (mm)	1.7 ± 0.03	1.8 ± 0.03	1.8 ± 0.05	1.7 ± 0.03
LVFS (%)	44.8 ± 0.8	43. 2 ± 0.6	40.9 ± 1.03^a^	45.3 ± 1.3 b
LVPWd (mm)	1.37 ± 0.05	1.5 ± 0.05	1.3 ± 0.02	1.5 ± 0.03^b^
LVPWs (mm)	1.52 ± 0.04	1.57 ± 0.03	1.41 ± 0.04	1.57 ± 0.03^b^

Heart rate (HR), left ventricular end diastolic (LVEDD) and end systolic (LVESD) diameters in female wild type (FWT) mice or female overexpressing aldosterone synthase (FAS) after 9  weeks postsurgery (Sham‐operation [Sham] or ovariectomy [Ovx]). Data are the means ± SEM.

*P* ≤ 0.05 versus FWT‐Sham.

a *P* ≤ 0.008 FAS‐Sham versus FWT‐Sham.

*P* ≤ 0.05 versus FAS‐Sham.

b *P* ≤ 0.006 FAS‐Ovx versus FAS‐Sham.

**Figure 2:**
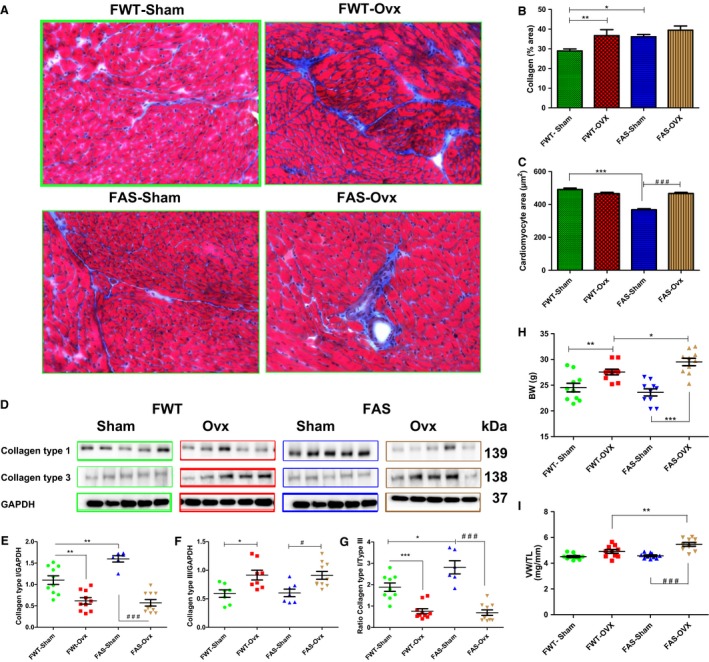
Histological changes and gravimetric parameters of the sham‐operated (Sham) or ovariectomized (Ovx) female wild type (FWT) or overexpressing cardiac aldosterone synthase (FAS) mouse hearts**.** (A) representative of bright‐field micrographs (magnification, 20×) of Masson's trichrome‐stained cardiac frozen sections from female sham‐operated or ovariectomized mice of both genotypes show that the collagen fibers were stained blue and the nuclei stained black and the cytoplasm was stained red. **(**B,C) collagen content was calculated as a ratio of the fibrotic area (blue staining) to total left ventricular myocardial area (blue and red staining); histomorphometric analysis as bar diagrams, show that aldosterone stimulated cardiac fibrosis; Ovx significantly increased collagen content and cardiomyocyte area in mice of both genotypes. (D) shows representative immunoblots of cardiac collagen type 1 or collagen type 3 protein expressions; (E,F) quantification of each collagen type immunoblots. The values expressed as mean ± SEM and normalized to GAPDH expression protein level (*n* = 5–10 in each group) (G) represents the collagen type I to collagen type III.*P < 0.05; **P < 0.001; ^#^ *P* < 0.05; and ^###^ *P* < 0.001. (G,H) gravimetric data from all experimental animal groups; (G) ovx induced a gain of body mass; (H, I), gravimetric data of body weight (H) ventricular hypertrophy index (I) calculated as the ratio of ventricle weight (VW) to tibia length (TL). *P < 0.05;**P < 0.01; ***P < 0.001, and ^###^ *P* < 0.001.

Gravimetric data are summarized in Table 2. The effect of Ovx was confirmed by the uterine atrophy in all Ovx mice. Estrogen depletion induced a significant increase in body weight (BW) in both genotypes. However, BW was markedly higher in the FAS‐Ovx mice than their in FWT‐Ovx mice (Fig. 2H). Figure 2 illustrates cardiac structural changes. Representative of bright‐field micrographs of Masson's trichrome‐stained cardiac frozen sections from all mice atnine weeks postsurgery are shown in Figure 2A. Collagen, myocytes, and nuclei are stained blue, red, and black, respectively. The image shows morphological evidence of heterogeneity in cardiomyocyte size (ranging from hypertrophied to atrophied) and collagen deposition (interstitial and perivascular, (blue staining)). The hearts of FWT‐Sham mice showed an intact and homogenous histoarchitecture. We observed some changes in myocardial architecture in the hearts of FAS‐ Sham mice. Histomorphometric examinations show a significant increase in collagen content. FAS‐sham mice exhibited higher collagen content than FW‐Sham mice (Fig. 2B). In addition, size cardiomyocyte area was also significantly reduced in FAS‐Sham mice (Fig. 2C) as compared with FWT‐Sham mice suggesting that aldosterone might be involved in cellular volume control. Representative immunoblots illustrated cardiac collagen type1 or type 3 protein expression and their respective quantification (Fig. 2D–F). FAS‐sham mice exhibited higher collagen type 1 protein level than FWT‐sham (Fig. 2E) while Ovx induced a downregulation of collagen type 1 protein level in female mice of both genotypes. Myocardial collagen type 3 exhibited a different pattern of expression than collagen type 1 in OVX mice. Both FWT‐ and FAS‐sham mice expressed similar collagen type 3 protein level (Fig. 2F). Female Ovx mice of both genotypes had a significant increase in collagen type 3 protein levels versus sham mice (*P* < 0.05). Ovx groups exhibited a significant decrease of collagen type I/Type III ratio (Fig. 2G). These quantitative subtype measurements of myocardial collagen by western blot revealed an isoform shift as collagen type III was the only significantly increased subtype after ovariectomy. Moreover, estrogen depletion induced a dual regulation of collagen. Increased collagen content was associated with Ovx‐induced collagen type3 upregulation. Estrogen depletion also induced an increase in hypertrophy index as the ratio of ventricle weight to tibia length that was significantly marked in FAS‐Ovx mice than in FWT‐Ovx mice (Fig. 2I).

Noninvasive blood pressure measurements in vigil mice are reported in Table 3. Conscious FWT and FAS sham groups exhibited similar blood pressure and no significant difference in heart rate was found. Ovx significantly induced increases in blood pressure (systolic and diastolic) and in heart rate in FWT‐Ovx mice. FAS‐Ovx mice exhibited a significant increase in heart rate, only.

**Table 2:** Gravimetric parameters of female WT or AS mice at 9 weeks postsurgery

Genotype	FWT		FAS	
Surgery	Sham	Ovx	Sham	Ovx
Number of mice	10	10	10	10
BW (g)	24.5 ± 0.8	27.6 ± 0.6^a^	23.6 ± 0.7^a^	29.6 ± 0.7^b^
HW (mg)	111.0 ± 5.3	106.5 ± 4.0	119.0 ± 4.0	114.8 ± 5.9
VW (mg)	84.4 ± 3.9	88.1 ± 2.7	95.1 ± 8.4	96.8 ± 3.3
AW (mg)	5.1 ± 0.4	5.8 ± 0.2	6.9 ± 0.6	6.7 ± 0.3
TL (mm)	18.2 ± 0.1	18.3 ± 0.1	18.1 ± 0.3	18.1 ± 0.1
UW (mg)	25.6 ± 0.9	28.0 ± 2.3	51.4 ± 0.2	22.5 ± 1.5
HW/BW (×10^−3^)	4.2 ± 0.2	3.9 ± 0.1	4.1 ± 0.2	3.9 ± 0.1
HW/TL (mg/mm)	5.6 ± 0.3	5.8 ± 0.2	5.7 ± 0.4	6.2 ± 0.3

FWT, female wild‐type mice; FAS, female mice overexpressing cardiac aldosterone synthase; Sham, sham‐operated; Ovx, bilateral ovariectomy; BW, body weight; HW, heart weight; AW, auricle weight; Tl, tibia length; UW, uterus weight. Data are the means ± SEM.

*P* ≤ 0.05 versus FWT‐Sham.

a *P* ≤ 0.001 FAS‐Ovx versus FWT‐Sham,

b *P* ≤ 0.006 FAS‐Ovx versus FAS‐Sham.

**Table 3:** Blood pressure measured by tail‐cuff plethysmography in vigil female WT or AS mice at 9 weeks postsurgery

Genotype	FWT		FAS	
Surgery	Sham	Ovx	Sham	Ovx
Number of mice	5	5	5	5
Systolic BP (mmHg)	119 ± 2	144 ± 5^a^	116 ± 4	108 ± 2^b^
Diastolic BP (mmHg)	79 ± 4	101 ± 6^a^	76 ± 3	78 ± 3^c^
Mean BP (mmHg)	91 ± 4	119 ± 5^a^	90 ± 3	89 ± 2^b^
Heart rate (bpm)	634 ± 20	759 ± 34^a^	536 ± 38	695 ± 38^b^

FWT, female wild‐type mice; FAS, female mice overexpressing cardiac aldosterone synthase; Sham, sham‐operated; Ox, bilateral ovariectomy; BP, blood pressure; Data are the means ± SEM.

*P* ≤ 0.05 versus FWT‐Sham.

*P* ≤ 0.001 versus FWT‐Sham.

a *P* ≤ 0.0001 FWT‐Ovx versus FWT‐Sham.

*P* ≤ 0.05 FAS‐Ovx versus FWT‐Ovx.

b *P* ≤ 0.001 FAS‐Ovx versus FAS‐Sham.

c *P* ≤ 0.0001 FWT‐Ovx versus FAS‐Ovx.

### Estrogen depletion downregulates the cardiac estrogen receptor protein expression

**Figure 3:**
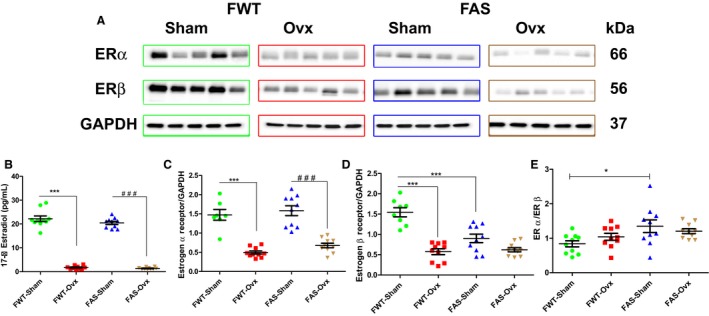
Effects of hyperaldosteronism and/or hypoestrogenism on estrogen status in female WT or AS mice. (A) shows representative immunoblots of cardiac estrogen receptor (ERα, ERβ) protein expression. (B) bar graphs show biochemical analysis of circulating 17‐β estradiol (E2) level in sham and Ovx mice. As expected, hypoestrogenism was induced by ovariectomy (Ovx) in female mice of both genotypes. (C, D), Ovx downregulated ER protein expression levels in females of both genotypes. (E) the ratio of (ERα, ERβ) protein isoform. The values expressed as mean ± SEM and normalized to GAPDH expression protein level (*n* = 5–10 in each group).*P < 0.05; ***P < 0.001; ^#^ *P* < 0.05; and ^###^ *P* < 0.001.

Representative immunoblots illustrated classical cardiac estrogen receptor (ER) protein expression and their respective quantification (Fig. 3A, C–E). Both estrogen receptors (ER*α* and ER*β*) were expressed in cardiac left ventricles of females mice both genotypes (Fig. 3C–D). Female Sham mice exhibited a significant higher cardiac ER*α* protein expression level than female Ovx‐ mice (Fig. 3C) while the ER*β* protein level was significantly downregulated in FAS‐Sham as compared with FWT‐Sham mice (Fig. 3D). Combined effects of estrogen depletion and elevated plasma aldosterone accentuated the downregulation of ER*β* protein level.

Although the uterine atrophy was measured (Table 2), the 17‐*β* estradiol (E2) levels were also evaluated in all animal groups to determine the hypoestrogenism induced by Ovx (Fig. 3B). As expected, Ovx significantly reduced the E2 level in Ovx mice of both genotypes (Fig. 3B). Circulating plasma E2 concentration dropped to values similar to those detected in male mice of similar age (MWT: 1.67 ± 0.21 pg/mL; MAS: 2.2 ± 0.58 pg/mL), suggesting other sources of E2 than gonadal source. Moreover, the Ovx‐induced hypoestrogenism markedly induced a significant downregulation of both cardiac ER*α* and ER*β* receptors in FWT mice as well as in FAS mice.

### Ovx modulated mineralocorticoid receptor expression and circulating plasma level of aldosterone

**Figure 4:**
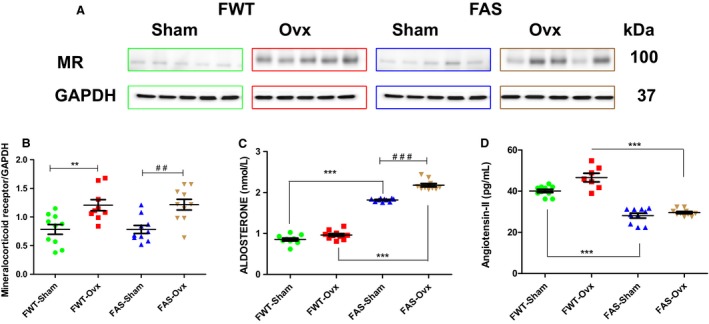
Effects of hyperaldosteronism and/or hypoestrogenism on circulating aldosterone level in female wild type (FWT) or overexpressing cardiac aldosterone synthase (FAS) mice. (A) shows representative immunoblots of cardiac mineralocorticoid receptor (MR) protein expression. (B) quantification of MR immunoblots. The values expressed as mean ± SEM and normalized to GAPDH expression protein level. (C) represents plasma aldosterone level (nmol/L), measured by radioimmunoassay. As expected, FAS mice exhibit a twofold increase in circulating aldosterone level. Ovx increased circulating plasma aldosterone in female mice of both genotypes. The values expressed as mean ± SEM (*n* = 5–10 in each group). **P < 0.01; ***P < 0.001; ^##^ *P* < 0.01; ^###^ *P* < 0.001.

As previously shown for male AS mice (Garnier et al. 2004), female AS mice also exhibited a twofold increase in plasma aldosterone level as compared with female WT (Fig. 4C). Representative immunoblots illustrated cardiac mineralocorticoid (MR) protein expression and their respective quantification (Fig. 4A and B). Estrogen depletion induced a significant increase in cardiac MR protein expression level in FAS mice as well as in FWT mice (Fig. 4B). Circulating plasma aldosterone level is higher in FAS‐Ovx mice then in FAS‐sham mice (Fig. 4C).

### Effects of hyperaldosteronism and/or hypoestrogenism on Ca^2+^‐handling proteins

**Figure 5:**
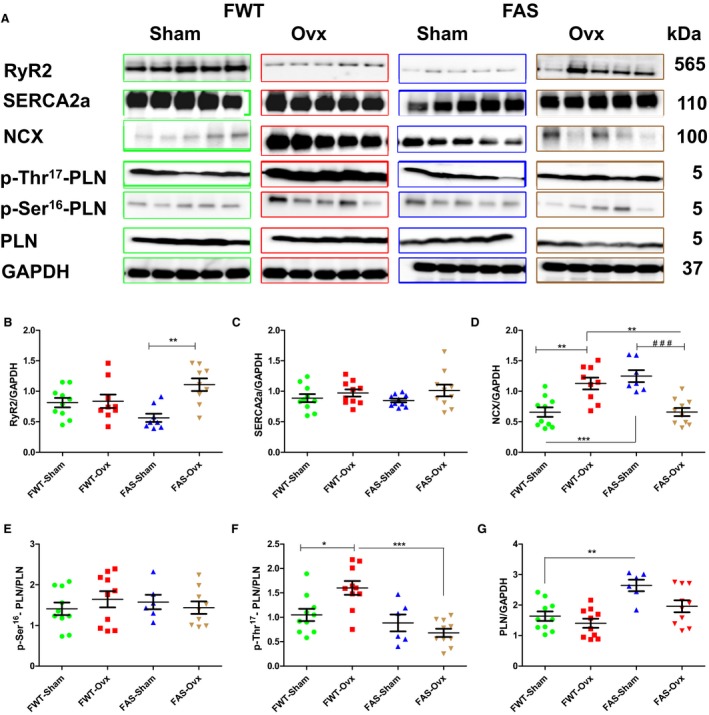
Effects of hyperaldosteronism and/or hypoestrogenism on calcium‐handling regulatory proteins in wild type (WT) or aldostereone synthase overexpressing (AS) mouse hearts**.** (A) shows representative immunoblots of ryanodine receptor (RyR2), sarcoplasmic reticulum Ca^2+^‐ATPase (SERCA2a), phospholamban (PLN) and its phosphorylation (Thr17 or Ser16), and Na^+^/Ca^2+^‐exchanger (NCX). (B–G) illustrate their respective quantification. The values expressed as mean ± SEM and normalized to GAPDH expression protein level (*n* = 5–10 in each group).*P < 0.05; **P < 0.01, and ^###^ *P* < 0.001.

Figure 5 represents immunoblots and quantifications of the major Ca^2+^ handling protein expression levels involved in the calcium homeostasis such as ryanodine receptor (RyR2), sarcoplasmic‐endoplasmic calcium‐ATPase 2 (SERCA2a) and its inhibitor phospholamban (PLN), sodium/calcium exchanger (NCX) in female sham or Ovx mice of both genotypes. RyR2 protein expression level was significantly downregulated in FAS‐Sham mice as compared with FWT‐Sham mice (Fig. 5B). We observed a dual effect of Ovx on RyR2 protein expression levels, either downregulated in FWT mice or upregulated in FAS mice. No significant difference in SERCA2a protein expression level was found in both genotype mice (Fig. 5C). NCX protein expression levels was twofold increased in FAS‐Sham mice as compared with FWT‐Sham mice (Fig. 5D). Ovx also exerted a dual effect on NCX protein expression: an upregulation, and a downregulation of NCX protein level in FWT and FAS mice, respectively. An increase in phosphorylation of PLN at Thr17 was found in FWT‐Ovx mice whereas a decrease was found in FAS‐Ovx mice. We observed a concomitant antagonistic effect of hypoestrogenism on RyR2 and NCX protein expression levels in genotype‐dependent manner.

### Effects of hyperaldosteronism and/or hypoestrogenism on activation of Ca^2+^‐signaling pathway (CaMKII/Cn/NFAT)

**Figure 6:**
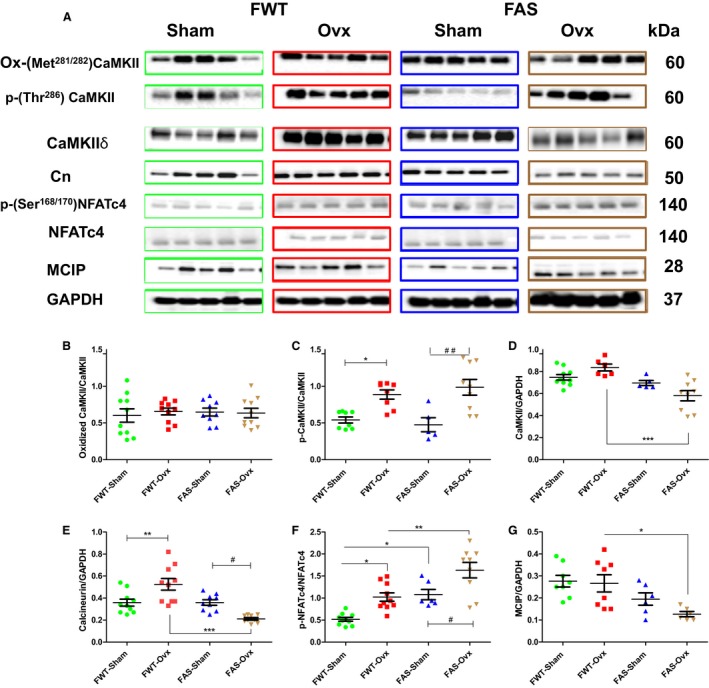
Effects of hyperaldosteronism and/or hypoestrogenism on activation of Ca^2+^‐signaling pathway (Cn/NFAT/CaMKII,) proteins in wild type (WT) or aldostereone synthase overexpressing (AS) mouse hearts. (A) shows representative immunoblots of activation of calcium‐calmodulin‐dependent kinase II (CaMKII): its oxidation (Ox‐CaMKII) or its phosphorylation (phospho‐Thr ^286^), and calcineurin (Cn), and nuclear factor of activated T‐cells (NFATc4) and its phosphorylation (phospho‐Ser^168/170^), and its inhibitor, calcipressin (MCIP). (B–G) illustrated their respective quantifications, CaMKII activity calculated as the ratio of Ox‐CaMKII to CaMKII, and/or p‐CaMKII to CaMKII. The ratio of p‐NFATC_4_ to NFATC_4_ indirectly reflected Cn activity. CaMKII and pan Cn and MCIP protein expression level normalized to that of GAPDH. The values expressed as mean ± SEM (*n* = 5–10 in each group). *P < 0.05; **P < 0.01; ***P < 0.001; ^#^ *P* < 0.05, and ^##^ *P* < 0.01.

A kinase, Ca^2+^ calmodulin‐dependant protein kinase II (CaMKII) and a phosphatase, calcineurin (Cn), both activated by Ca^2+^‐calmodulin play a major role in the transmission of the hypertrophic signals to cell nucleus. Representative immunoblots of active forms of CaMKII (phospho‐Thr^286^: p‐CaMKII, and methionine 281/282 oxidation: ox‐CaMKII) are shown in Figure 6(A–D). Both the active form of CaMKII were found in the heart. No significant difference in oxidation of CaMKII (ox‐CaMKII) protein level was found in female mice of both genotypes (Fig. 6B). However, Ovx induced an increase in CaMKII activation: phospho‐CaMKII was twofold increased in Ovx mice of both genotypes.

Immunoblot analysis of total calcineurin (Cn) and its inhibitor calcipressin (MCIP) and phosphorylation of NFATc4 status shows in Fig. 6 (A, E–G). It revealed that female WT or AS mice expressed similar Cn protein level. No significant difference in MCIP protein expression was found between sham‐mice of both genotypes. Ovx induced a significant upregulation and downregulation of the Cn protein expression level in FWT and FAS mice, respectively. The phosphorylation status of NFATc4 indirectly reflected Cn activity. When Cn is active, it dephosphorylates NFATc4 allowing its nuclear translocation. As expected, the lowest Cn protein expression level in FAS‐Ovx mice was also associated with higher phosphorylated NFACTc4 protein level therefore blocking its nuclear translocation. In FWT mice, Ovx upregulated Cn protein expression level and it was associated with lower phosphorylated NFATc4.

### Effects of hyperaldosteronism and/or hypoestrogenism on activation of MAP Kinases (ERK1/2, p38‐MAPK, and c‐Jun)

**Figure 7:**
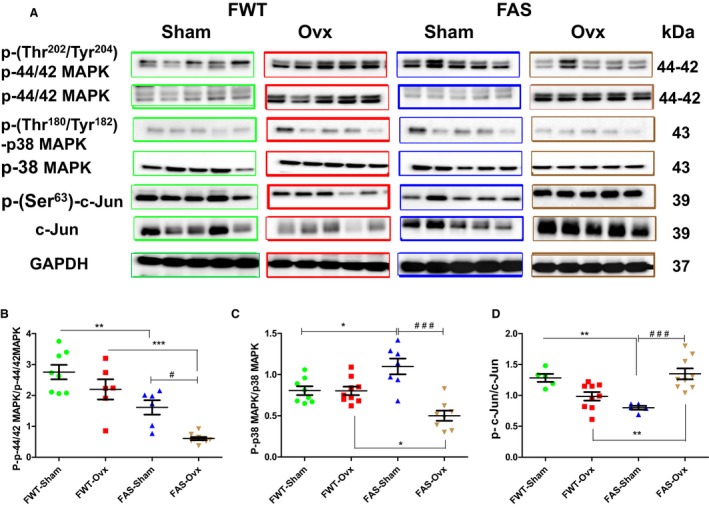
Effects of hyperaldosteronism and/or hypoestrogenism on selected MAP Kinase activation proteins (ERK1/2, p38‐MAPK, c‐Jun) in female wild type (FWT) and aldostereone synthase overexpressing (FAS) mouse hearts. (A) shows, respectively, immunoblots of the phosphorylated and total forms of each mitogen‐activated kinases (phospho‐Thr ^202^/Tyr ^204^‐ERK1/2; ERK1/2 (p‐44/42 MAPK); and phospho‐Thr^180^/Tyr^182^p38MAPK; p38 MAPK; and phospho‐Ser^63^‐c‐Jun; c‐Jun). (B–D) corresponded to their respective quantification as the ratio of phosphorylated form to total form. Immunoblot of glyceraldehyde 3‐phosphate dehydrogenase (GAPDH) served as loading protein control. The values expressed as mean ± SEM (*n* = 5–10 in each group). *P < 0.05, **P < 0.01; ^#^ *P* < 0.05, and ^###^ *P* < 0.001.

Representative immunoblots and quantification of phosphorylation of ERK1/2 (p42/44‐MAPK) and p38‐MAPK, c‐Jun; (phospho‐Tyr^202^/Y^204^: p‐Erk1/2; phospho‐Thr^180^/Tyr^182^: p‐p38‐MAPK; phospho‐Ser^63^: p‐cJun p39) are shown in Figure 7A–D.

Elevated circulating aldosterone significantly reduced by twofold ERK1/2 phosphorylation in FAS mice as compared with FWT mice, but ERK1/2 activation persisted in FAS‐Sham mice. Ovx also reduced the activation of ERK1/2. Hypoestrogenism drastically accentuated the inhibition of ERK1/2 in FAS mice (Fig. 7B). Aldosterone significantly increased p38 MAPK phosphorylation in FAS‐Sham mice as compared with FWT‐Sham mice (Fig. 7C). Ovx did not affect p38 MAPK phosphorylation status in FWT mice whereas it was markedly decreased in FAS‐Ovx mice (Fig. 7C). Aldosterone significantly decreased by 2.5 fold c‐Jun phosphorylation in FAS‐Sham mice as compared to FWT‐Sham mice (Fig. 7D). No significant activation of c‐Jun was observed between FWT‐Sham and FWT‐OVX mice. However, FAS Ovx mice significantly exhibited higher (1.5 fold) c‐Jun phosphorylation level than FWT‐Ovx mice. In addition, c‐Jun activation was significantly higher in FAS‐Ovx mice than FAS‐Sham mice. Taken together, these results suggest that MAPK activation in response to aldosterone was antagonized by hypoestrogenism.

### Effects of hyperaldosteronism and/or hypoestrogenism on pro/anti‐ hypertrophic Signaling (Akt/GSK3β)

**Figure 8:**
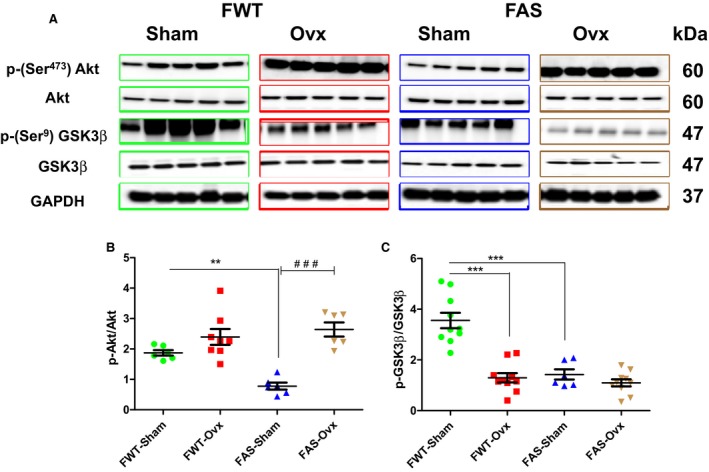
Effects of hyperaldosteronism and/or hypoestrogenism on pro/anti hypertrophic signaling proteins (Akt/GSK3β) in female wild type (FWT) or aldostereone synthase overexpressing (FAS) mouse hearts. (A) shows representative immunoblots of phospho‐Akt (p‐Ser^473^ Akt, active form), Akt, phospho‐GSK3β (p‐Ser^9^ GSK3β), GSK3β (active form), and glyceraldehyde 3‐phosphate dehydrogenase (GAPDH). (B and C) quantification of Akt and GSK3β phosphorylation were expressed as the ratio to Akt (B) and to GSK3β (C), respectively. Immunoblot of glyceraldehyde 3‐phosphate dehydrogenase (GAPDH) served as loading protein control. The values expressed as mean ± SEM (*n* = 5–10 in each group). **P < 0.01, *** and ^###^ *P* < 0.001.

Immunoblots of the LV protein levels and phosphorylation status of Akt (phospho‐Ser ^473^, active form) and GSK3*β* (phospho‐Ser^9^, inactive form) are illustrated in Figure 8. We measured the ratio of phosphorylated to total Akt and GSK3*β* (Fig. 8B–C, respectively) as a measure of kinase activity in the heart. After comparing the ratio of active to total Akt, it is clear that E2 depletion increased Akt active form in female of both genotypes (Fig. 8B) FWT‐Sham mice exhibited higher level of Akt active form than FAS‐Sham mice, associated with the predominant GSK3*β* inactive form. Although Ovx induced Akt activation, it maintained the balance between the two forms of GSK3*β* (Fig.8Χ)

### Effects of hyperaldosteronism and/or hypoestrogenism on SR/ER stress (GRP78, PDI, CHOP)

**Figure 9:**
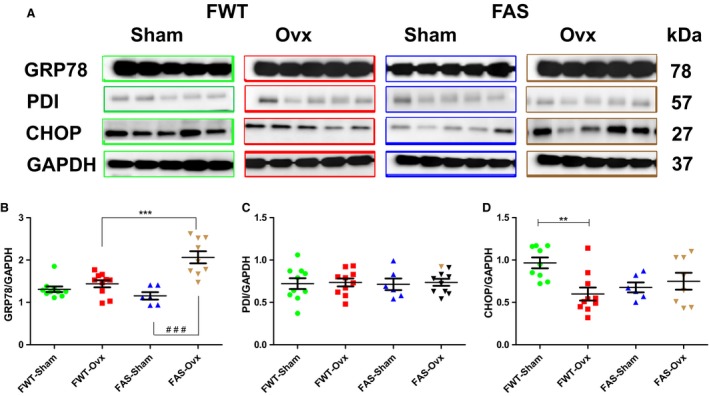
Effects of hyperaldosteronism and/or hypoestrogenism on few selected SR/ER stress proteins in female wild type (FWT) or aldostereone synthase overexpressing (FAS) mouse hearts. (A) shows representative immunoblots of GRP78, PDI and CHOP. (B–D) reported the quantification of each targeted proteins and their protein expression level normalized to those of GAPDH. The values expressed as mean ± SEM (*n* = 5–10 in each group. **P < 0.01, ***P < 0.001, and ^###^ *P* < 0.001.

Cardiac 78‐kDa glucose–regulated protein (GRP78) also known as BiP, protein disulfide isomerase (PDI) and CHOP also known as growth‐arrest‐and DNA damage inducible gene153 (GADD153) used as indicators ER stress condition, were assessed by Western blot. Immunoblots and quantification are shown in Figure 9A–D. GRP78 protein expression level was significantly higher in FAS‐Ovx mice than FWT‐Ovx mice (Fig. 9B) whereas their respective controls exhibited similar protein level. Ovx induced a twofold increase in GRP78 protein expression level in FAS‐OVX‐mice as compared with FAS‐Sham mice. PDI protein expression level was unchanged whatever experimental conditions (Fig 9C). No significant difference in CHOP protein expression level was found between FWT and FAS mice. However, Ovx significantly induced a downregulation of CHOP protein expression in female WT mice, only (Fig. 3D).

### Effects of hyperaldosteronism and/or hypoestrogenism on pro/anti apoptotic signaling

**Figure 10:**
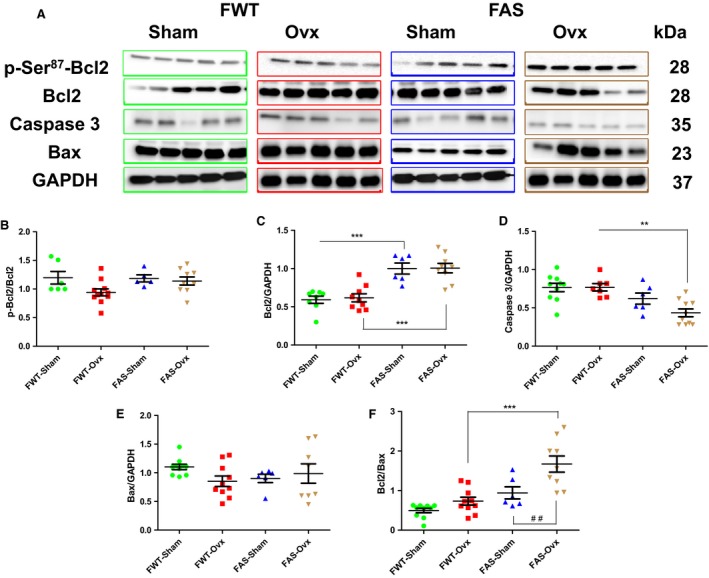
Effects of hyperaldosteronism and/or hypoestrogenism on selected pro/anti‐apoptotic signaling molecules in female wild type (FWT) or aldostereone synthase overexpressing (FAS) mouse hearts. (A) shows representative immunoblots of phospho‐Bcl2 (phospho‐Ser^87^), Bcl2, caspase 3 and Bax. (B–E) reported their respective quantification. The phosporylation of Bcl2 was calculated as the ratio of phosphorylated form to total form (B), and, Bcl2 (C), caspase 3 (D), and Bax (E) protein expression levels were normalized to those of GAPDH. (F) the ratio of Bcl2 to Bax indicates the balance between pro‐ or antiapoptotic signals. The values expressed as mean ± SEM (*n* = 5–10 in each group). **P < 0.01; ***P < 0.001; and ^##^ *P* < 0.01.

Immunoblots of the two LV key protein levels regulating cellular apoptosis: the anti‐apoptotic Bcl2 and its phosphorylation status (phospho‐Ser^87^) and the pro‐apoptotic Bax, and the caspase 3 are illustrated in Figure 10A–F. No significant difference in Bcl2 activation (p‐Bcl2) was found between FWT and FAS mice (Fig. 10B), but FAS mice exhibited higher Bcl2 protein expression level than FWT mice. Ovx did not change Bcl2 protein expression profile in FAS mice (Fig. 10C). Ovx decreased by twofold caspase 3 protein expression levels in FAS‐Ovx mice (Fig. 10D). No cleavage of caspase 3 was observed in all animal groups. No significant difference in Bax protein expression level between FWT and FAS mice (Fig. 10E). The ratio Bcl2/Bax is in favor to promote cell survivor (Fig. 10F). The absence of proteolytic processing of caspase 3 reinforced the apoptosis repressor Bcl2 activity.

### Effects of hyperaldosteronism and/or hypoestrogenism on redox signaling

**Figure 11:**
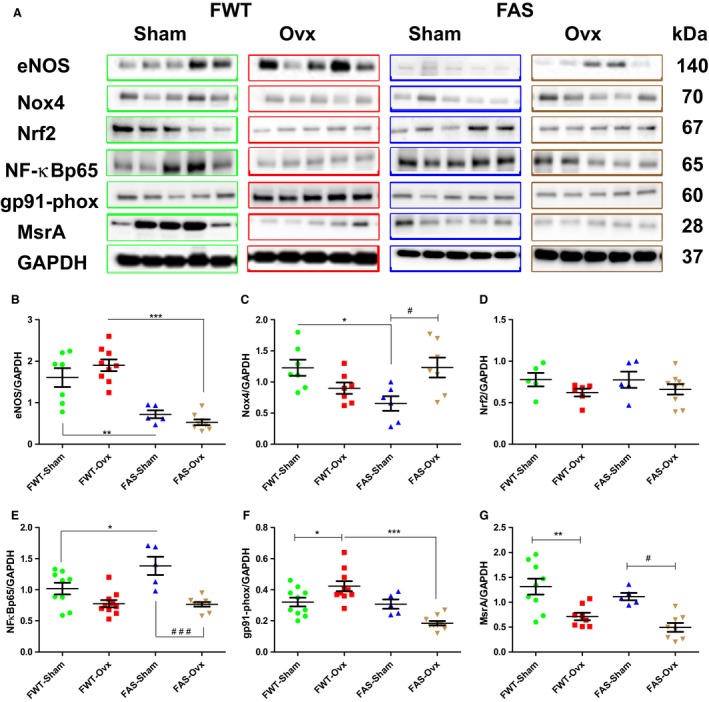
Effects of hyperaldosteronism and/or hypoestrogenism on redox molecule signaling in female wild type (FWT) or aldostereone synthase overexpressing (FAS) mouse hearts. (A) shows representative immunoblots of endothelial nitric oxide synthase (eNOS), NADPH oxidases (gp91‐phox, Nox4), transcription factors (Nrf2 and NF‐κ p65) and methionine sulfoxide reductase (MsrA). (B–G) illustrated their respective quantification. Each protein expression level was normalized to that of GAPDH. The values expressed as mean ± SEM (*n* = 5–10 in each group). *P < 0.05; **P < 0.01; ***P < 0.001, ^#^ *P* < 0.05, and ^###^ *P* < 0.001.

We focused on some protein targets involved in the redox signaling such as endothelial nitric oxide synthase (eNOS) producing NO; NADPH oxidases (gp91‐phox as oxygen sensor, subunit of Nox2 superoxide‐producing; Nox 4 constitutively active producing stable signaling molecule hydrogen peroxide (H_2_O_2_) with a longer half‐life than superoxide) and two main transcription factors associated with the response to oxidative stress, as NF‐*κ*B involved in the development of cardiac hypertrophy, and redox–sensitive transcription factor Nrf2 mediating the induction of anti‐oxidant enzymes; methionine sulfoxide reductase (MsrA reversing oxidation of methionine in proteins). These myocardial protein expressions were assessed by Western blot and are illustrated in Figure 11. Elevated circulating aldosterone induced a significant marked decrease in protein expression levels of eNOS, Nox4, gp91‐phox in FAS‐Sham mice as compared with FWT‐Sham mice (Fig. 11 B, C and F). No significant difference in Nrf2 protein expression level was found in all animal groups (Fig. 11D). E2 depletion significantly downregulated the activated NF‐*κ*B (i.e., its p65 subunit) protein levels in FAS‐Ovx mice (Fig. 11E). Female Sham mice of both genotypes expressed higher MsrA protein levels than Ovx mice. Ovx significantly induced a downregulation of MsrA protein in female mice of both genotypes (Fig. 11G).Taken together these data suggest that E2 and aldosterone shared similar targeted molecules of the redox signaling and had antagonized effects.

## Discussion

In the present study, hyperaldosteronism and/or Ovx‐induced hypoestrogenism murine models were used to assess a comparative integrated insight into the changes that occurred with myocardial remodeling and functional compensatory adaptations to these chronic injuries in female transgenic AS or WT mice. Having in mind, myocardial responses are biological and multifactorial processes, therefore our data from cardiac phenotype reflected some subtle dynamic spatiotemporal molecular, cellular and interstitial changes including a mismatch between the different roles of various cell types (cardiomyocytes, endothelial cells, fibroblasts, pericytes and other mesenchymal cells, macrophages and other leukocytes) and signaling factors in regulating the repair of injured myocardium. The major findings are that

1. As expected, hyperaldosteronism was associated with higher circulating aldosterone level (twofold) in FAS mice than in FWT mice, but similar to those of male AS mice (Garnier et al. 2004). FAS‐sham mice had lower heart rate and FS than FWT‐sham mice. Aldosterone lowered the circulating Ang‐II plasma level. In contrast with FWT‐Sham mice, aldosterone induced a reduction of cardiomyocyte size accompanied with an increase in collagen content in FAS‐Sham mice. Aldosterone differentially modulated the cardiac estrogen receptor protein expression in FAS‐Sham mice. Aldosterone modified the profile of Ca^2+^‐handling protein expression, MAP kinase activation, and redox signaling.
2. As expected, Ovx induced hypoestrogenism in female mice of both genotypes associated with uterine atrophy. Moreover, it drastically dropped circulating plasma 17‐*β* estradiol (E2) level, but low level of E2 was still detected in female mice and found similar to those detected in male mice suggesting other gonadal sources. Both cardiac estrogen receptors (ER*α*, ER*β*) protein expression levels were downregulated in Ovx mice. Both sham mice expressed similar cardiac MR protein level. Ovx similarly induced upregulation of MR protein level. Interestingly, Ovx increased circulating aldosterone in FAS mice, only. Ovx increased gain weight and cardiac hypertrophy index. In addition, hypoestrogenism induced collagen sutbtype isoform shift to collagen type III.
3. Both hyperaldosteronism and hypoestrogenism induced an increase in body and cardiac ventricle weight. They slightly increased fractional shortening used an estimate of myocardial contractility that may be influenced either by heart rate and/or the action/response of the sympathetic nervous system as a result of catecholamine release. Both hormone levels and their respective cardiac receptor protein expression are altered. Both insults differentially influenced the expression of multiple signaling effectors of extracellular matrix (ECM), calcium/redox homeostasis, apopotsis and on hypertrophic signaling pathway. Here, they induced a collagen shift. In FAS mice, hypoestrogenism played as mirror effect of hyperaldosteonism on targeted signaling molecules: such as altered Ca^2+^‐handling proteins (RyR2 upregulation and NCX downregulation), enhanced the MAPK inhibition, and stimulated the pro‐survival signaling pathway (Akt activation). Therefore, hypoestrogenism antagonized or counterbalanced some effects of hyperaldosteronism.

### Effects of hyperaldosteronism on myocardial phenotype in female AS mice

FAS‐sham mice exhibited twofold increases in circulating aldosterone as compared with FWT mice corroborating the previous results observed in males (Garnier et al. 2004). Aldosterone acts centrally to stimulate sympathetic tone and to increase the blood pressure (Leenen, 2014; Joëls and de Kloet 2017; Tasic and Lovic 2018). Despite its poor penetration, circulating aldosterone can readily access neurons in brain nuclei outside the blood brain barrier, which express MR and induce an increase in sympathetic nerve activity. Moreover, the progressive hypertension caused by a chronic increase in circulating aldosterone can be prevented by specific blockade. Using a selective MR antagonist, such as eplerenone, aldosterone blockade reduces MI‐induced cardiac remodeling and phenotypic alterations of gene expression preferentially in females than in males (Kanashiro‐Takeuchi et al. 2009). It has also been reported that acute and chronic peripheral aldosterone administration impairs baroreceptor responses in dogs (Wang 1994) and in humans (Monahan et al. 2007). MR activation is also involved in reducing hypothalamic‐pituitary–adrenocortical (HPA) axis activity (Murck et al. 2014). In addition, microinjection of aldosterone into nucleus ambiguus decreases the heart rate in conscious rats and produces bradycardia (Brailoiu et al. 2013). In regard with these observations, lower heart rate and slight decrease in fractional shortening (FS) observed in anesthetized FAS mice may reflect either potential interference of anesthetics with the cardiovascular reflex or the bradycardic response induced by elevated circulating aldosterone. If the ventricle does not fill normally during diastole the FS% will be reduced. FS% is particularly sensitive to changes in afterload. An increase in systemic blood pressure or an increase in myocardial stiffness will therefore reduce FS%. However, FS% can also be influenced by the heart rate. In addition, FS slightly reduced which might be due to the increase of collagen type I protein expression level.

Previous studies indicate that elevated levels of aldosterone promote fibrosis, leading to renal and cardiovascular dysfunction (Lavall et al. 2014; Pruthi et al. 2014). Among the pleiotropic aldosterone actions (Brown 2013), in order to dissect the effects of aldosterone from the effects of systemic hypertension on the expression of profibrotic genes in the male heart, hypertensive global renin (Ren) transgenic mice were crossed with normotensive AS transgenic mice. Hypertension was associated with increased cardiac expression of genes encoding fibronectin, CTGF and TGF‐*β*1 and there was no difference in the cardiac expression of these profibrotic genes between Ren and Ren‐AS mice (Azibani et al. 2013). Sex differences play a significant role in the incidence of cardiovascular diseases. Aldosterone has also been shown to stimulate collagen and other extracellular matrix protein expression and secretion from cardiac fibroblasts (Brilla 2000). In contrast with FWT‐Sham mice, aldosterone induced an increase of collagen type I concomitantly associated with a reduction of cardiomyocyte size in FAS‐Sham mice. Corroborating with excess of aldosterone is well known to induce myocardial fibrosis (Brown 2013); and to be deleterious on heart (Catena et al. 2015; Kritis et al. 2016).

Moreover, estrogen receptor signaling is essential for sex hormone‐dependent cardioprotection against adverse remodeling. Both cardiac estrogen receptors were differentially expressed in female sham mice: the myocardial isoform ER*β* predominantly expressed in FWT‐Sham mice while FAS‐Sham mice exhibited higher ER*α* protein level than ER*β*. Several reports show that antagonistic action of ER*β* and ER*α* on proliferative effects of estrogen (Liu et al. 2002), and an important physiological role of ER*β* is to modulate ER*α*‐mediated gene transcription supporting a “Ying Yang” relationship between ER*β* and ER*α* in mice (Lindberg et al. 2003). Therefore, the imbalance of ratio of ER*α*/ER*β* in favor of ER*α* in FAS‐Sham mice might prevent the beneficial anti‐fibrotic effects of ER*β*. The circulating E2 level was similar in both genotype mice. Although the activation of ER*α*, ER*β*, or both provide cell protection by upregulating of p‐Akt, here, elevated circulating aldosterone was associated with an inactivation of Akt and a drastic downregulation of eNOS protein expression in FAS‐Sham mice. This simultaneous impairment would have been triggered cardiac dysfunction. In parallel, ERK1/2 activation, another downstream target of nonnuclear estrogen signaling, thereby promoting NO production in heart, might mediate compensatory effects on heart contractility. Moreover, aldosterone is well known to induce ROS generation and other ROS‐dependent kinases, such as CaMKII, are involved in aldosterone–mediated signaling (He and Anderson 2013). CaMKII has also been showed to play an important role in regulating the activity of both RyR2 and SERCA2a (Bers 2010), two major key Ca^2+^‐handling proteins involved in excitation‐contraction coupling. In addition, a higher level of oxidized CaMKII was observed in FAS‐Sham mice. This methionine oxidation of CaMKII result from oxidative stress generated from mitochondrial H_2_O_2_ (Erickson et al. 2011). It has been shown that H_2_O_2_‐mediated oxidation of methionine residues (Met 281/282) in CaMKII activates the kinase activity and phosphorylates PLN, and RyR2, rendering it leaky and thereby depleting the SR Ca^2+^ content. Circulating elevated levels of aldosterone have been reported to regulate L‐type calcium (Ca^2+^) currents in ventricular myocytes (Perrier et al. 2005) intracellular Ca^2+^ movement, and activation of the ryanodine receptor (Gómez et al. 2009) whereas mildly elevated levels of aldosterone have been found in patients with essential hypertension, and elevated levels trigger hypertrophy, fibrosis, and remodeling (Tsybouleva et al. 2004; Brilla et al. 1990; Young et al. 1994; Lin et al. 2012). A concomitant downregulation of RyR2 protein level and upregulation of NXC and PLN protein expression level without change in SERCA2a protein expression found in FAS‐Sham mice let us to speculate that increase in NCX protein level might counteract this aberrant calcium release and uptake tending to restore Ca^2+^‐homeostasis. NCX has also been shown to play a role in altered calcium handling and vascular dysfunction in hypertension (Goulopoulou and Webb 2014). Recent data showed that ER stress and Nrf2 activation reduce myocardial infarction and cardiac hypertrophy in animals upon pressure overload (Cominacini et al. 2015). In addition, we showed that oxidation of CaMKII occurred in heart upon high level of aldosterone, which can be counterbalanced by an increase in MsrA expression level (enzyme that reduced oxidized‐CaMKII) through Nrf2 activation. This data suggest that CaMKII activation is redox‐modulated in hyperaldosteronism. Aldosterone has both genomic effects, mediated by activation of traditional MR, and rapid nongenomic actions that are independent of transcription (Grossmann and Gekle 2009; Funder 2010). Moreover, MAPKs such as extracellular signal‐regulated kinases (ERK), c‐Jun NH_2_‐terminal kinases (JNK), and p38 MAPK play a pivotal role in transducing extracellular signal (Davis 1993) and their activities depending on their respective phosphorylation status to elicit a response by the cell. These components, are sensitive and quantitative markers for hypertrophic responses in heart (Arabacilar and Marber 2015) Aldosterone activated ERK1/2 and inhibited p38‐MAPK and c‐Jun in FAS mice. p38 MAPK coordinate the cellular responses needed for adaptation and survival and its role in cardioprotection has been recently reviewed (Martin et al. 2015). In parallel, aldosterone also activated NF*κ*B, a well‐known transcription factor, after its activation (NF*κ*Bp65), regulates variety of cellular biological effects such as inflammation, cell proliferation, cell survival and tissue growth (Donato et al. 2015; Ghosh and Dass 2016). Recently, NF*κ*B activation has pleiotropic effects on heart regeneration and is important for heart tissue repair (Karra et al. 2015). Taken together p38 MAPK inhibition and NF*κ*B activation may provide additional benefit by reducing the effects of myocardial inflammation and consequently improving in myocardial remodeling. In addition, the ratio of Bcl2 to Bax indicates that the balance between pro‐/antiapoptosis in favor of antiapoptotic Bcl2 pathway was reinforced by the absence of cleaved caspase3. These different findings suggest the presence of complicated signaling processes and the interactions involved in the myocardial remodeling. Therefore, heart has carefully managed balance between pro‐ and antihypertrophic and apoptotic and redox signals to maintain correct function in FAS‐Sham mice.

### Effects of hypoestrogenism on myocardial phenotype in female WT or AS mice

To study the effects of menopause on murine myocardium, we have used the bilateral ovariectomy model allowing a uniform estrogen deficiency in animals. We observed a body weight gain in female Ovx mice of both genotypes. Our findings are consistent with those of previous studies showing body mass gain in Ovx rodents (Pimenta et al. 2015) and in postmenopausal women (Grygiel‐Górniak et al., 2016). Naturally, three estrogens (estrone (E1), 17*β*‐estradiol (E2) and estriol (E3)) exit in a dynamic equilibrium in circulation. Of these, E2 is the most important sex hormone that protects against cardiovascular diseases in women (Dworatzek and Mahmoodzadeh 2017).

As expected, bilateral ovariectomy induced hypoestrogenism in female mice of both genotypes associated with uterine atrophy and a drastic dropped down E2 level. Moreover, the presence of low circulating level of E2 similar to those measured in male mice suggests that it is secreted by other nongonadal sources (brain, heart, liver; etc.). Menopausal women also exhibited serum E2 concentration similar to, or lower than those in men of similar age (Luo and Kim 2016). Additionally, it has been shown that men with abnormal level of E2 exhibited the highest death rates from congestive heart failure (Jankowska et al. 2009).

We only focused on classical estrogen receptor protein expression (ER*α* and ER*β*). E2 deficiency was accompanied with a drastic downregulation of both myocardial estrogen receptors (ER*α* and ER*β*). Recent studies suggest that ER*α* and ER*β* play each distinct role in cardioprotection and a greater role for ER*α* versus ER*β* in the modulation of endothelial progenitor cells and cardiac repair (Dawn and Bolli 2006; Hamada et al. 2006). Nine weeks after Ovx, ER*α* is the predominant cardiac subtype expressed in female mice of both genotypes. Although ER subtype distribution and activation were not assessed, the expression of both ER has been detected in the whole heart lysates, therefore we can speculate that myocardial ER*α* and ER*β* might mediate functional responses to low E2 in different cell types/cellular locations whilst other estrogen receptors such GPER could not be excluded. Concomitantly, Ovx induced an upregulation of myocardial MR expression in female mice of both genotypes. Although the immunodetection of myocardial MR expression level enabled us to demonstrate the presence of functional MR in heart, these in vivo observations are consistent with previous reports showing that E2 suppresses the synthesis and transactivation of the MR in brain and vascular endothelial cells (Barrett Mueller et al. 2014) and allowed us to speculate that E2 depletion and ER (*α* and *β*) downregulation might contribute to favor MR protein abundance. Further studies are necessary to confirm or to refute to the role of ER/MR complex in MR synthesis or default in MR degradation**.**

In the absence of high level of aldosterone, FWT‐Ovx mice exhibited a slight reduced heart rate without any changes in the other measured echocardiographic parameters. Ovx‐induced collagen type III shift might contribute to “normal” cardiac function However, low level of E2 alters the Ca^2+^‐handling protein expressions. Although functional studies of three key Ca^2+^‐handling proteins (RyR2, SERCA2a, and NCX) were not assessed here; we observed that concomitant changes occurred in heart, such as a downregulation of RyR2, no change in SERCA2a, and upregulation of NCX. PLN has been suggested to be a major regulator of cardiac contractility (Koss and Kranias 1996). Additionally, the posttranslational modification of PLN (phospho‐Thr^17^) resulted from Ca^2+^‐ CaMKII activation (phospho‐Thr^286^), which alleviates its inhibition on SERCA2a and might result in greater activation of Ca^2+−^ATPase affinity for Ca^2+^, which can be associated with an increase in contractility. Recently, CaMKII activation either by Ca^2+^ overload or oxidation, can contribute to pro‐apoptotic pathway (Feng and Anderson 2017). In parallel, Ovx induced up–regulation of eNOS and gp91–phox (Nox2 subunit) protein expression pathway in FWT mice, thereby contributing to increase the activity of pro‐oxidant generating. Although endogenous NO is essentially involved in many physiological processes and beneficial actions in a variety of circumstances, its reaction products may mediate nitrosative and oxidative stress (Stamler et al. 1992). Regarding the complexity of the regulation of the eNOS expression (Fleming 2010), an important role has been attributed to the transcription factor NF‐*κ*B (Balligand et al. 2009). Ovx induced a decrease in NF‐*κ*Bp65 protein level. Concomitantly, Ovx also leads to Akt activation by phosphorylating Akt at Ser^473^ in FWT. It is well recognized that Akt pathway promotes survival by downregulating pro‐apoptotic factors and upregulating antiapoptotic factor. Additionally, the increased Bcl2/Bax ratio observed in female Ovx mice supports the hypothesis that activated Akt pathway could be anti‐apoptotic and cardioprotective.

### Combined effects of hypoestrogenism and hyperaldosteronism on myocardial remodeling in female AS mice

The menopause in women is a natural process related with the loss of generative ovarian function, and with it, the loss of fertility and many somatic and psychological problems. Several studies have demonstrated that mineralocorticoid receptor blockers improve mood and reduce anxiety among human and animals (Johnson and Grippo 2006; Hlavacova and Jezova 2008). Aldosterone has also been considered as a potentially important mediator of the relationship between psychological stress and cardiovascular disease (Kubzansky and Adler 2010; Murck et al. 2014). Regarding these observations suggested crosstalk between aldosterone and estrogen signaling, but several reports are conflicting about it. MR and ER play important roles and may functionally interact in blood pressure maintenance and in the adaptive response of myocardium toward increase workload and/or injury. Hyperaldosteronism and ovariectomy‐induced hypoestrogenism were associated with a down–regulation of both estrogen receptors (ER*α* and ER*β*), and a downregulation of eNOS, suggesting that NO production might be low. These results are supported by similar findings in other groups (Förstermann et al.^1994^; Brede et al. 2003; Lin et al. 2008). Surprisingly, hypoestrogenism and hyperaldosteronism induced a significant moderated increase in MR and in circulating aldosterone level. These observations were consistent with recent report showing that silencing of the ER*β* significantly raised aldosterone synthase expression and aldosterone production in humans; E2 inhibits aldosterone synthesis by acting via ER*β* (Caroccia et al. 2014). Moreover, the increase in acute MI in postmenopausal women may be related to the decrease of estrogen in the body and decrease in the expression of ER*β* (Burns et al. 2011).

E2 loss and aldosterone excess also induced myocardial fibrosis (interstitial and perivascular). Of the main collagen types, the major fibrillar collagens are types I and III, which are essential components of the extracellular matrix (ECM), maintaining myocardium structural and functional integrity (Pauschinger et al. 1999). Therefore, myocardial collagen accumulation and collagen subtypes protein expression as assessed by histomorphometric analysis and by Western blotting revealed that hypoestrogenism inversed the collagen type I/collagen type III ratio. The increase in collagen type III counterbalanced the decrease in collagen type I in FAS‐Ovx mice. Additional studies are needed to clarify the mechanisms responsible for these collagen changes. Due to their different physical properties, this isoform collagen type III shift might confer greater elasticity and therefore have a major impact on the diastolic and systolic function of the heart. It has been recognized that altered collagen proportion was associated with depressed cardiac function in patients with dilated cardiomyopathy (Bishop et al. 1990; Marijianowski et al. 1995; Pauschinger et al. 1999), coronary artery disease (Wei et al. 1999), and with progression to heart failure in animal models (Mukherjee and Sen 1993; Woodiwiss et al.^2001^). Therefore, quality of collagen is an important factor in determining the degree of cardiac stiffness (Yang et al. 1997). Our data suggests that collagen shift seemed to be beneficial and might contribute to ameliorate cardiac function by increasing FS% in FAS mice. Additionally, previous reports showed that MAPK pathway participates to differential regulation of the collagen types I and III expression (Tang et al. 2008); age and estrogen deprivation following menopause alters the expression and activation of the MAPK family members p38 and ERK) in heart (Pinceti et al. 2016). In line with these studies, the decrease in collagen type I might be associated with the inhibition of ERK1/2 and p38 MAPK in FAS‐Ovx mice.

Additionally, the calcineurin/NFAT pathway is also known as a regulator of pathological cardiac remodeling as well as CaMKII signaling and their time‐dependent and isoform peculiarities on their role in cardiac hypertrophy and remodeling processes (MacDonnell et al. 2009). Calcineurin is involved in the development of cardiac hypertrophy and fibrosis induced by mineralocorticoid excess (Takeda et al. 2002).Another negative crosstalk between these two enzymes has also been suggested dephosphorylation of CaMKII at its autophosphorylation site by calcineurin (Kubokawa et al. 2011). Consistent with the complex interplay between these two enzymes, our data show that the downregulation of calcineurin protein level was accompanied with an increase of active form of CaMKII (phosphor‐CaMKII) in murine hypoestrogenism and hyperaldosteronism model.

In parallel, oxidation of CaMKII occurred in heart of FAS‐Ovx mice. The methionine oxidation of CaMKII result from oxidative stress generated from mitochondrial H_2_O_2_ (Erickson et al. 2011). Hypoestrogenism and hyperaldosteronism induced an increase in NADPH oxidase (Nox4) protein expression, known to act as physiological sensor of oxygen and generate hydrogen peroxide (H_2_O_2_) (Sun 2007). Therefore, Nox4 might lead to a subsequent ROS generation, in turn could also participate to the oxidation of CaMKII. He et al. (2011) have also reported that CaMKII is activated in myocytes by aldosterone which induced CaMKII oxidation by recruiting NADPH oxidase. Concomitantly, a weak Nrf2 protein expression was associated with a downregulation of MsrA protein expression which in turn could lesser reverse CaMKII oxidation caused by aldosterone‐induced ROS. Therefore, at molecular level, CaMKII activation (phospho‐ and/or ox‐CaMKII) was accentuated in FAS‐Ovx mice. These posttranslational modifications of CaMKII activate the kinase activity and phosphorylate PLN, and RyR2, rendering it leaky and thereby depleting the SR Ca^2+^ content. We did not find any change in SERCA2a protein level. SERCA2a expression and activity could be induced during ER stress in cardiomyocytes and other cells, suggesting a potential role of SERCA2a protein in ER stress. In addition, an increase of NCX protein level might counteract this aberrant calcium release and uptake to restore Ca^2+^‐homeostasis in FAS–Ovx mice. However, this local calcium imbalance in SR might have an effect on endoplasmic reticulum (ER) stress. The ER is a multifunctional organelle responsible for the synthesis and folding of proteins as well as for signaling and calcium storage that has been linked to the contraction‐relaxation process (Berridge 2002). Upon persistent ER stress, cells undergo pro‐apoptotic rather than pro‐adaptive pathways, which result in higher expression of apoptosis‐related genes such as CHOP and Caspase‐3 (Santos et al. 2009; Hetz 2012). However, we showed that OVX and excess of aldosterone caused an upregulation of GRP78 protein, a master regulator of ER signaling pathway, and reduced cellular apoptosis by increasing expression of Bcl2 and decreasing the apoptotic factors CHOP and caspase 3. Consistent with previous studies that overexpression of chaperone proteins such as PDI, GRP78, XBP1 and thrombospondin protects cardiomyocytes from ER stress as well as improving cardiac function (Kitakaze and Tsukamoto 2010; Toldo et al. 2011; Wang et al. 2017). Moreover, Akt signaling, a central molecule of cellular survival mechanisms, phosphorylates many downstream targets, including glycogen synthase kinase 3*β* (GSK‐3*β*) and Bcl‐2 family (Hers et al. 2011). GSK‐3*β* phosphorylation is a step to which multiple protective signaling pathways converge. GSK‐3*β* phosphorylation‐dependent modulation of mitochondrial Bcl2 has been demonstrated (Das et al. 2008; Juhaszova et al. 2009). Bcl‐2 family proteins are regulated by reversible phosphorylation modifications that control their activity and conformation (Haldar et al., 1995; Shitashige et al. 2001). Phosphorylated Bcl‐2 is known to be predominantly localized within the ER and the mitochondrial membrane (Ruvolo et al. 1998; Estaquier et al. 2012). As anticipated from calcineurin enzymatic loss (downregulation of Cn), phosphorylated Bcl‐2 might also promote cell survival through the stabilization of the mitochondrial membrane. In the scenario, heart upon hypoestrogenism and hyperaldosteronism, Akt activation enhanced phosphorylation of GSK‐3*β* with a subsequent phosphorylated Bcl‐2 and an increase in the Bcl2/Bax ratio, thereby increasing the anti‐apoptotic activity of Bcl2. As a whole, dual signaling of hypoestrogenism and hyperaldosteronism mediate these alterations which are thought to favor (promote) myocardial plasticity and restore cardiac function in FAS‐Ovx mice.

## Conclusions

In summary, hypoestrogenism conferred opposing effects on hyperaldosteronism‐induced myocardial phenotypes. Elevated circulating aldosterone plasma levels in combination with low circulating E2 plasma levels promoted cardiac remodeling including myocardial modified Ca^2+^‐proteins, collagen homeostasis, and anti‐apoptotic phenotype, and moderate cardiac hypertrophy. Although the in situ myocardial bioavailability of E2 and aldosterone and the subsequent estrogen and/or mineralocorticoid receptor activation could not be distinguished, we showed that they are differentially expressed in the heart in response to high aldosterone levels. Despite OVX‐induced downregulation of both ER receptors (ER*α* and ER*β*) and upregulation of MR, nevertheless, it conceivable that low E2 and ER subtypes might drive function in protecting the cardiovascular system against the detrimental effects of aldosterone. Therefore, from these in vivo studies, it is plausible that the dynamic interplay between SR/ER stress, MAPK signaling, Ca^2+^‐proteins homeostasis, apoptotic and redox molecule signaling reflected the pleiotropic responses of cardiac myocytes and other myocardial cells (EC, VSMC, fibroblasts, etc.,) without excluding other complementary and antagonistic actions of signaling cascades might contribute to maintain cardiac function.

Finally, better elucidation of ER and MR localization, and mechanisms in gene transcription, intracellular signaling, function and intercellular communication, and the interactions among NO/ROS/RN production in the heart in situ will offer new targets to develop novel therapeutic approach.

## Limitation

Our hypoestrogenism and hyperaldosteronism mouse model is an integrative physio/pathological animal model, and the process of elucidating the various myocardial ER and MR pathways is complicated by the crosstalk between genomic and nongenomic ER and MR signaling as many genomic activities are both regulated by nongenomic signaling and can subsequently influence nongenomic pathways. This study was performed on myocardial tissue postmortem, therefore, the molecular dynamic of different signaling pathways were frozen at this time. However, many questions remain unanswered and/or incomplete. What degree these receptors mediate functional response to different circulating estrogen and aldosterone in different cell types and/or cellular locations. How the heart orchestrates these complex interrelations between the ubiquitous mechanisms integrate into other signaling pathways essential for cell survival and stress responses. Our results show that both low circulating E2 level and ER (*α*,* β*) downregulation are sufficient to counteract aldosterone‐altered signaling pathways and to restore myocardial homeostasis. In view of this, more studies are also needed to fully understand the precise mechanism by which hypoestrogenism mediates its beneficial effects under hyperaldosteronism.

## Conflict of Interest

All authors declare that they have no conflict of interest.
